# Advanced and flexible multi-carrier receiver architecture for high-count multi-core fiber based space division multiplexed applications

**DOI:** 10.1038/srep27465

**Published:** 2016-06-08

**Authors:** Rameez Asif

**Affiliations:** 1 Centre of Photonic Systems (CPS), Electrical Engineering Division, Department of Engineering, University of Cambridge, 9 J.J Thomson Avenue, Cambridge, CB3 0FA, United Kingdom

## Abstract

Space division multiplexing (SDM), incorporating multi-core fibers (MCFs), has been demonstrated for effectively maximizing the data capacity in an impending capacity crunch. To achieve high spectral-density through multi-carrier encoding while simultaneously maintaining transmission reach, benefits from inter-core crosstalk (XT) and non-linear compensation must be utilized. In this report, we propose a proof-of-concept unified receiver architecture that jointly compensates optical Kerr effects, intra- and inter-core XT in MCFs. The architecture is analysed in multi-channel 512 Gbit/s dual-carrier DP-16QAM system over 800 km 19-core MCF to validate the digital compensation of inter-core XT. Through this architecture: (a) we efficiently compensates the inter-core XT improving Q-factor by 4.82 dB and (b) achieve a momentous gain in transmission reach, increasing the maximum achievable distance from 480 km to 1208 km, via analytical analysis. Simulation results confirm that inter-core XT distortions are more relentless for cores fabricated around the central axis of cladding. Predominantly, XT induced Q-penalty can be suppressed to be less than 1 dB up-to −11.56 dB of inter-core XT over 800 km MCF, offering flexibility to fabricate dense core structures with same cladding diameter. Moreover, this report outlines the relationship between core pitch and forward-error correction (FEC).

## State of the art

With the advent of new smart phones, tablets and several communicating devices, we are entering in cloud computing era. The advanced mobile communication systems deployed around the globe are based on 4G or LTE technology. Next generation services such as cloud computing, 3D HDTV and Internet-of-Things (IoT) require unprecedented optical channel bandwidths. High speed global traffic is increasing at a rate of 30–40% every year^1^. Due to this reason, we are getting caught in bandwidth capacity crunch^2^. In order to maintain the quality of service (QoS), researchers are paying attention to backbone optical networks^3^,^4^,^5^. In recent years, numerous experiments have demonstrated the increment in spectral efficiency of fiber networks^6^,^7^,^8^,^9^. These include the use of orbital angular momentum (OAM)^10^,^11^, multi-mode based multiple-input multiple-output transmission (MIMO)^12^,^13^ or either by implementing advanced modulation formats^14^,^15^, such as m-ary quadrature amplitude modulation (QAM) and orthogonal frequency division multiplexing (OFDM)^16^,^17^,^18^. The major limitation, of OAM and multi-mode systems, is reduced transmission distance due to intra-core XT, mode coupling and mode gain equalization that is a major issue for multimode EDFA. Hence, conventional fiber networks are still favored commercially and researchers are investigating either by optimizing the fiber link design^19^,^20^,^21^ or by incorporating digital signal processing (DSP) to compensate the fiber impairments^22^,^23^,^24^,^25^,^26^,^27^.

Coherent detection is considered efficient along with DSP to compensate many linear effects in fiber propagation i.e. chromatic dispersion (CD). It also offers low required optical signal-to-noise ratio (OSNR). Despite of CD and non-linearities which are the major limiting factors, optical transmissions are employing multi-carrier higher order modulation formats in order to increase the spectral efficiency^28^. As a result, compensation of CD and non-linearities (NL), i.e. self-phase modulation (SPM), cross-phase modulation (XPM) and four-wave mixing (FWM), is a point of high interest from past few years^29^. On the other hand, multi-carrier signal transmission has attracted much attention^30^,^31^. Most recently, systems with sub-carrier spacing less than the baud-rate have been reported achieving super high density and effectively compensating XT among the neighboring sub-carriers using MIMO^32^,^33^.

Recently, SDM has been a prominent choice of researchers^34^ to use multiple parallel paths for data transmission, i.e. MCFs^35^. Record transmission capacity of 2.15 Pbit/s^36^ and 2.05 Pbit/s has been demonstrated^37^. As compared to few-mode-fiber (FMFs)^38^,^39^,^40^,^41^, MCFs can provide a smooth transition from existing SMF networks to SDM due to the availibity of off-the-shelf transceivers^42^. To achieve >Pbit/s or Ebit/s transmission, the future road-map leads to the manufacturing of ultra dense cores in a single cladding^43^,^44^. The MCFs also have relatively low SDM XT as compared to modal XT in FM-MCFs where transmission has thus far been limited to ≈500 km^45^. The MCFs structure inherits wavelength dependent linear XT, depending on core-to-core distance (termed as core pitch ‘Λ’), that is negligible for fewer number of cores, i.e. 7- or 12-core fibers^46^,^47^. This effect limits the maximum number of cores that can be placed over 210 *μ*m or 230 *μ*m of cladding diameter^48^. While there are concentric efforts being made to reduce the XT by adopting different techniques, i.e. trench-assisted structure, heterogeneous core arrangement and propagation-direction interleaving, therein-after termed as all-optical design methods^49^,^50^,^51^. There have been few demonstrations investigating the NL impairments in MCFs in digital domain^52^,^53^ and evaluating the inter-core differential mode delay characteristic in coherent transmission^54^. A brief summary of different MCF types according to their number of cores and structures are enlisted in Table 1.

However, to date a comprehensive coherent receiver architecture for high-count MCFs transmission has not been investigated. Yet in MCFs based multi-carrier multi-channel transmission, the impact of fiber non-linearities are more significant as the channel spacing among the channels is reduced. While the use of digital backward propagation (DBP) algorithm along with coherent receiver may give considerable benefits by compensating the intra- and inter-channel non-linear effects^55^,^56^,^57^. Still intra-core XT from the neighboring sub-carriers and inter-core XT from dense core geometry of the MCFs can degrade the long-haul transmission performance significantly. Therefore, it is indispensable to study detailed coherent receiver architecture that can compensates the above mentioned factors to enhance the practicability of long-haul MCFs networks.

In this report, for the first time to the best of our knowledge, we propose a novel unified multi-carrier receiver architecture for long-haul MCF-SDM transmission. This receiver design is capable of jointly and digitally compensating: (a) the optical Kerr effects using digital backward propagation (therein-after termed as DBP), (b) intra-core XT among the neighboring sub-carriers using extended Kalman filter based MIMO equalizer (therein-after termed as XTC) and (c) linear wavelength dependent inter-core XT using least mean square equalizer (therein-after termed as IXTC). The primitive receiver model is analytically evaluated in 32 Gbaud multi-channel 512 Gbit/s dual-carrier DP-16QAM transmission system (corresponding to an aggregate 29.1 Tbit/s capacity and 1.5 Tbit/s/core data rate). The fiber link comprises of 10 spans (80 km per span of fiber) of 19-core MCF with 210 *μ*m cladding diameter emulating a distance of 800 km supported by a standard multi-core Erbium doped fiber amplifier (MC-EDFA). The performance of dual-carrier DP-16QAM signal is investigated in detail to quantify the performance in the presence of strong inter-core XT. To evaluate the receiver complexity the system is further evaluated in-terms of achievable transmission distance and Q-factor, by opting different receiver scenarios, i.e. (a) only compensating optical Kerr effects (DBP), (b) compensating optical Kerr effects with intra-core XT among the neighboring sub-carriers (DBP + XTC) and (c) incorporating the DSP module to compensate wavelength dependent inter-core XT (DBP + XTC + IXTC). The analytical results have confirmed efficient receiver performance by post-processing the inter-core XT, likewise exhibit full-flexibility for future deployment of optical networks incorporating high-count MCFs.

## Transmission model and DSP architecture

**Figure 1:**
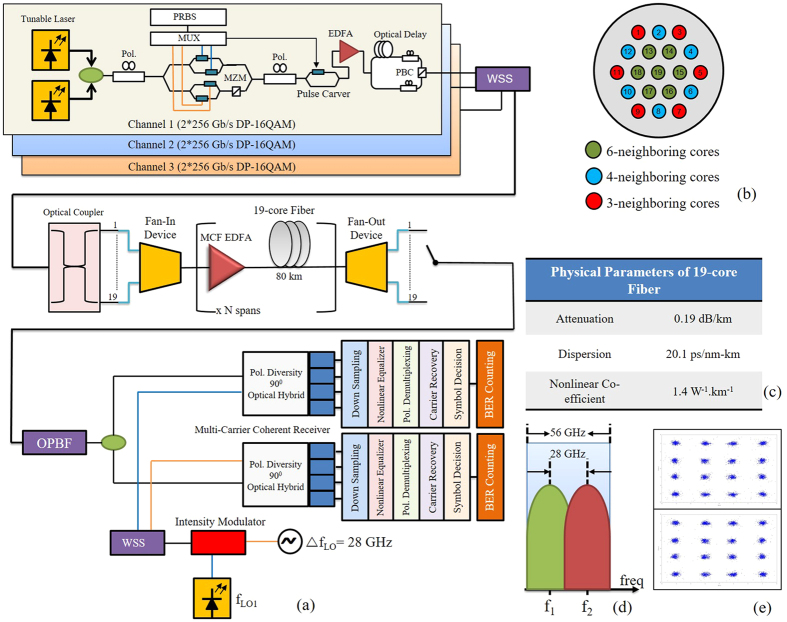
(**a**) Schematic of multi-channel 512 Gbit/s dual-carrier DP-16QAM transmission system with 19-core MCF and DSP, (**b**) Core arrangement of the 19-core MCF, (**c**) Physical properties of the 19-core MCF, (**d**) Channel spacing in sub-carriers of DP-16QAM signals and (**e**) Recovered constellation diagrams of the sub-carriers in back-to-back configuration. [Pol: polarization, MUX: Multiplexer, PRBS: pseudo random binary sequence, MZM: Mach-Zehnder modulator, PBC: polarization beam combiner, WSS: wavelength selective switch, OPBF: optical band-pass filter, EDFA: Erbium doped fiber amplifier].

Figure 1(a) depicts the model of long haul SDM transmission. A pseudo-random binary sequence (PRBS) of length 2^15^ − 1 is modelled with distributed feedback laser (DFB) bank that is emulated with 100 kHz line-width to generate WDM channels with 28 GHz channel spacing achieving a 32 Gbaud 512 Gbit/s dual-carrier DP-16QAM (56 GHz bandwidth per dual-carrier channel), as in Fig. 1(d). The sub-carrier spacing is 28 GHz corresponding to 87% of the signal baud rate. The transmitted symbol sequences are de-correlated using a cyclic time shift by 170 symbols before being combined and polarization multiplexed. Three dual-carrier signals were multiplexed with central channel sub-carriers at 1550 nm and 1549.77 nm wavelength. The signals were then split into 19 identical copies through optical couplers. The impact of polarization mode dispersion (PMD) is considered negligible in analysis. A 90° polarization and phase diversity homodyne coherent receiver is used to detect the signals, with no bandwidth limitation allowing for the ideal detection of in-phase and quadrature components of both the polarization states in our simulations. The bit-error rate (BER) threshold was set to be 3.8 × 10^−3^ (Q-factor of 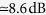), corresponding to a 7% overhead hard-decision forward error correction (HD-FEC). Electronic dispersion compensation (EDC) is implemented by using a frequency-domain-based static equalizer. While polarization de-multiplexing is achieved using a time-domain based adaptive equalizer (*T*/2-spaced taps with butterfly configuration), optimized by a decision-directed (DD) least mean-square (LMS) algorithm (a constant modulus algorithm (CMA) is used for pre-convergence). Furthermore, root-raised-cosine (RRC) filter with a roll-off of 0.1% is used for the Nyquist pulse shaping (NPS). Carrier frequency and phase recovery algorithms are implemented with-in the DD-LMS equalization loop. For jointly compensating the high speed signals, homodyne signal detection is a requisite^31^. To generate XT among the sub-carriers two dual-polarized signals are optically coupled with the sub-carrier spacing of Δ*f* = *f*_2_ − *f*_1_, which is less than the system baud-rate.

**Figure 2:**
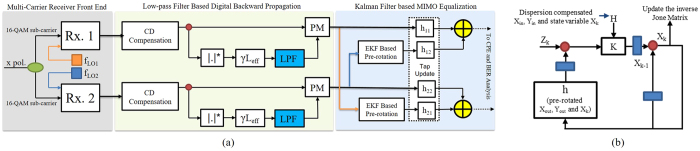
(**a**) High capacity multi-carrier transmission model with digital backward propagation and extended Kalman filter based adaptive MIMO equalization and (**b**) extended Kalman filter flow-chart.

The modelled communication link comprises of a 19-core MCF having attenuation (*α*), chromatic dispersion (*β*) and non-linear coefficient (*γ*) of 0.19 dB/km, 20.1 ps/nm-km and 1.4 W^−1^.km^−1^ at 1550 nm, respectively, as shown in Fig. 1(b,c). We have considered 800 km transmission distance by emulating 10 spans of 80 km each. Back-to-back system performance is analyzed and the constellation diagrams the sub-carriers are depicted in Fig. 1(e). The core arrangement of the 19 cores gives us 3 different sets of cores according to their neighbors, i.e. 6-,4- and 3-neighboring cores. The cladding diameter (CD) is considered as 210 *μ*m while core pitch Λ is 35 *μ*m. We have used the analytical model as depicted in^50^ to measure the wavelength dependent intra-core XT of every core. To summarize, after 800 km of transmission the core 19 has the XT value of −16 dB. We have incorporated an in-line MC-EDFA in the model to emulate the compensation of fiber attenuation. The MC-EDFA is modelled with a gain factor of 15.2 dB and noise figure of 6 dB^58^. The received multi-carrier signals are processed through homodyne detection using local oscillators (LOs) where the frequency spacing between the LOs is Δf_*LO*_ = f_*LO*2_ − f_*LO*1_, as depicted in Fig. 2(a). DSP in the system incorporates the functions of DBP, extended Kalman filter (EKF) based MIMO equalization, and carrier phase recovery (CPR). The DBP algorithm is implemented by a non-iterative asymmetric split-step Fourier method (A-SSFM)^27^ incorporating a low-pass-filter (LPF) in-order to diminish the out of band distortions^59^. For the compensation of intra-core XT among the neighbouring sub-carriers, an EKF based adaptive MIMO equalization is applied along with DBP, as depicted in Fig. 2(b). After the SOP estimation and pre-rotation, adaptive MIMO equalization has 31-tap *T*/2-spaced FIR filters and the tap coefficients are updated using CMA.

**Figure 3:**
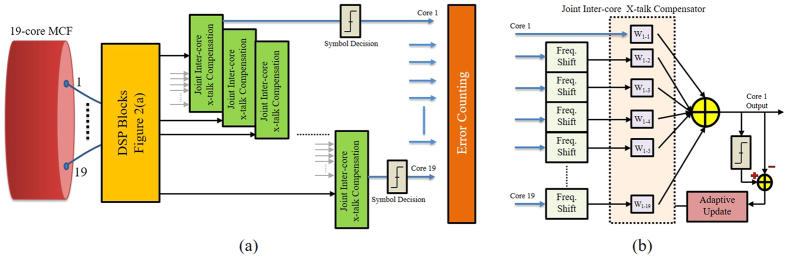
(**a**) Joint DSP architecture to compensate the inter-core XT based on adaptive LMS algorithm supported by DSP modules of DBP and carrier XT compensator and (**b**) flow-chart of the adaptive LMS equalizer.

We further proposed the DSP architecture of LMS algorithm^60^, as depicted in Fig. 3(a), to compensate the inter-core XT. After processing through the DSP blocks as depicted in Fig. 2, the adaptive LMS equalizer is applied across all the cores. Whereas, the filter coefficients (*w*_11_, *w*_12_, *w*_13_ and so on) are jointly and adaptively updated. Each filter coefficient *w*_*i*,*j*_ represents the weighted cross-talk from core 1 to 19, as shown in Fig. 3(b). The number of taps depends on the timing off-set as well as the XT value according to the number of neighbors between the cores, it is found that 9 to 21 taps are usually sufficient. The joint inter-core XT compensation algorithm is quite flexible and can be applied: (a) to any type of modulation format (e.g. DP-32QAM, DP-64QAM) assuming other parts of the DSP algorithm are compliant and (b) to any number of fiber cores in the MCF. All the numerical analysis are done via VPI Transmission Maker v9.2 while mode-solver and DSP algorithms are implemented in Matlab v.2015.

## Analytical Results

**Figure 4:**
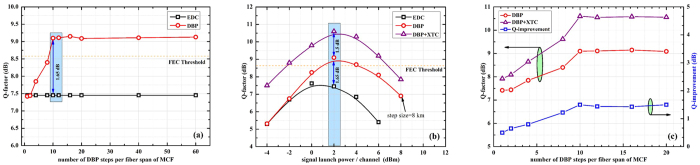
(**a**) Complexity comparison of DBP algorithm as a function of number of computational steps per MCF span, (**b**) Calculated signal quality of the dual-carrier signal as a function of signal input power after 800 km dispersion uncompensated MCF link without DBP (only linear equalization (EDC)), with DBP and DBP + XTC and (**c**) Compatibility comparison of DBP and XTC.

The back-to-back performance of the transmitter is initially analysed using the middle dual-carrier signal and the constellation diagrams are illustrated in previous section. Further, we quantify the performance of system by using EDC and DBP after 800 km 19 core MCF link. The performance of the signals in 19^*th*^ core of the MCF are discussed here, as this core is intensely affected by the XT. In order to achieve the maximum performance from DBP, we initially optimize the number of calculation steps per fiber span. The analytical results are depicted in Fig. 4(a). At 2 dBm signal launch power, EDC gives the Q-factor of 7.45 dB, whereas the Q-factor after the post-processing of signals via DBP gradually increases with step-size. At 10 calculations steps per fiber span (step size of 8 km) we get the maximum Q-factor, i.e. 9.1 dB. If we increase the step size further there is no significant improvement in system performance. In the following analysis, the DBP is implemented with 10 steps per span and the non-linear coefficient of 1.4 W^−1^.km^−1^ to ensure optimum operation of non-linear compensation. We vary the signal launch power from −4 dBm to 8 dBm. The results are plotted as in Fig. 4(b). It can be seen that when only EDC is applied, the non-linear threshold (NLT) point is at 0 dBm and at higher power the performance is dominated by non-linear effects, hence Q-factor deteriorates. When the signals are processed by DBP non-linear effects are efficiently compensated and the results depicted two-fold benefit, i.e. improvement in the NLT point by 2 dB with an associated Q-improvement of 1.65 dB. As we are operating in multi-carrier regime, so signal is also affected by intra-core XT. To compensate this impairment, we have implemented an EKF based MIMO equalizer, i.e. XTC. We confirm that MIMO based XTC in combination with DBP improves the after-transmission Q-factor, as depicted in Fig. 4(b), by 1.5 dB when it is compared to the system performance when only DBP is applied. We like to mention that for a system with only XTC, the Q-improvement decreases as fiber launch power increases. We assume that fiber non-linearity reduces the improvement obtained by XTC because the neighboring crosstalk does not dominate the Q-impairment in the higher power region. In high power region where fiber non-linearity impairs the signal quality, we already confirmed that NLC improves the Q-factor. So XTC should be used in combination with DBP especially for higher order modulation formats and for long haul transmission. Thus DBP enhances the system performance and extends the applicability of super high density multi-carrier transmission systems to the higher-power region. The compatibility of DBP and XTC is depicted in Fig. 4(c). At 2 dBm signal launch power, if we increase the number of computations for DBP the performance of XTC also improves. We can also conclude from these results that the non-linear phase shift accompanied with DBP does not deteriorate the performance of XTC.

**Figure 5:**
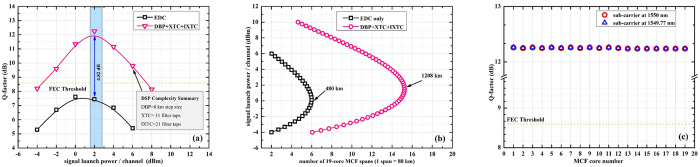
(**a**) Calculated signal quality of the dual-carrier signal as a function of signal input power after 800 km dispersion uncompensated MCF link with DBP + XTC + IXTC algorithm, (**b**) Comparison of distance versus launch power at BER of 3.8 × 10^−3^ and (**c**) Signal quality of all the simulated data tributaries of 19-core MCF.

The performance of IXTC in the multi-carrier DP-16QAM transmission system has also been investigated in terms of Q-factor for different optical launch powers, as shown in Fig. 5(a). This algorithm is applied to the received signals in combination with XTC and DBP. The system performance is compared with the EDC. The best system performance from the proposed receiver architecture, i.e. DBP + XTC + IXTC, is obtained at 2 dBm signal launch power and we have attained a Q-factor of 12.27 dB. These results clearly depicts a Q-improvement of 4.82 dB. It is discernible that for long-haul densely packed high-count MCFs, having inter-core XT compensation in DSP is imperative. It will not only increase the transmission reach of the network but also provides flexibility to fabricate more core in the same cladding diameter, i.e. further reduction of Λ. Having established the performance improvements available from proposed receiver design, in the next analysis we investigate the maximum attainable distance by dual-carrier MCF transmission, employing DBP + XTC + IXTC. The results are depicted in Fig. 5(b) and we plotted the achievable transmission distance at FEC threshold 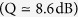. We achieve 480 km and 1208 km by employing EDC and DBP + XTC + IXTC, respectively in a 19-core MCF. We can infer from these results (enhancement of transmission distance by a factor of 3×) that IXTC is equally important as optical Kerr effects compensation for long-haul MCF transmission. However we evaluate the maximum reach at BER of 3.8 × 10^−3^ and assuming that additional FEC margin will be utilized for system margin allocation, otherwise soft-decision FEC (SD-FEC) level of BER 2.1 × 10^−2^ can also be used corresponding to 20% overheard. Furthermore, we have analytically measured all data tributaries from all 19 cores of the MCF after DSP. The results as depicted in Fig. 5(c) show that all the data tributaries are successfully detected above the FEC threshold.

## Discussion

**Figure 6:**
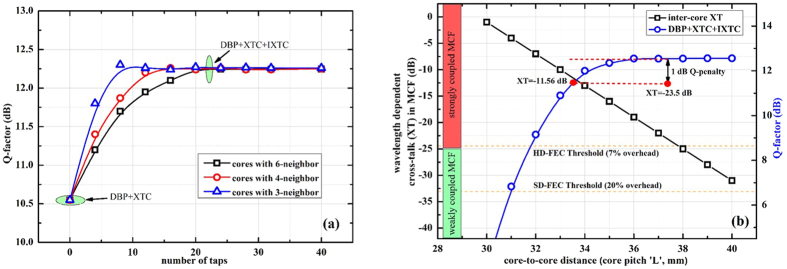
(**a**) Complexity of adaptive LMS algorithm with reference to the XT impact on the cores and (**b**) Measured core-to-core distance (Λ) w.r.t. the signal quality and XT implementing SD- and HD-FEC limits.

It is worth mentioning that wavelength dependent inter-core XT is directly related to the number of neighboring cores. The more neighbors a core has the greater is the impact of XT on it. We have considered and investigated a 19 core MCF in this report. Due to the geometry of the cores, this fiber has 3 sets of XT values. Among all these sets we have considered the condition of homogeneity. After the transmission distance of 800 km the XT values are: −16 dB, −17.95 dB and −19.1 dB for cores having 6-, 4- and 3- neighboring cores, respectively. The results in previous section depicted that XT can be efficiently compensated for improved transmission performance. This DSP module comprises of N × N matrix (where N corresponds to the number of cores), considering a single polarization, and in this analysis LMS algorithm is optimized with 21-filter taps. The Fig. 6(a) shows the complexity in-terms of filter taps for each individual core. We can see from the results that the cores with less number of neighboring cores can converge on optimum Q-factor using less number of filter taps. Therefore to simplify the DSP further, we recommend that in case of densely packed high-count fiber the cores in the outer ring can be neglected based on their XT value. The transmission penalty is negligible, if the XT values are −16.7 dB for DP-QPSK, −23.7 dB for DP-16QAM, and −29.9 dB for DP-64QAM^61^,^62^. Based on these values MCF is categorized as weakly coupled and strongly coupled fiber. This receiver architecture has wider applications for strongly coupled MCFs. Moreover, it will be helpful to implement the simplified DSP in real-time networks to avoid off-line processing or to reduce latency.

Another indispensable benefit from the proposed receiver architecture is that we can fabricate densely packed high-count MCFs by reducing Λ. A comparison of proposed receiver performance and Λ is given in Fig. 6(b). In this analysis, we have investigated the behavior of multi-carrier signals when Λ is gradually reduced and hence increasing the inter-core XT. The input power is kept constant at 2 dBm that is the NLT point of the system. As a reference we choose −23.7 dB of XT^61^ and measured the system performance. The corresponding Q-factor was 12.55 dB with 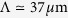. According to our mathematical model with every 1 *μ*m decrease in Λ, the XT value is increased by 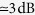. Through the post-processing of inter-core XT by our proposed receiver the Q-penalty can be suppressed to be less than 1 dB till the XT value of −11.56 dB. It can also be seen from the results that by opting HD-FEC processing the Λ can be reduced to 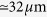 corresponding to −7.45 dB XT value after 800 km transmission. Similarly, SD-FEC limit can be opted to reduce Λ to 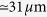. It should be kept in mind that our all XT calculations and analysis are based on 210 *μ*m cladding diameter. Based on the fact that we can compensate the inter-core XT via DSP, we can opt for different all-optical design methods, i.e. square-lattice, double-ring structure^49^, to incorporate more cores in a single cladding.

**Figure 7:**
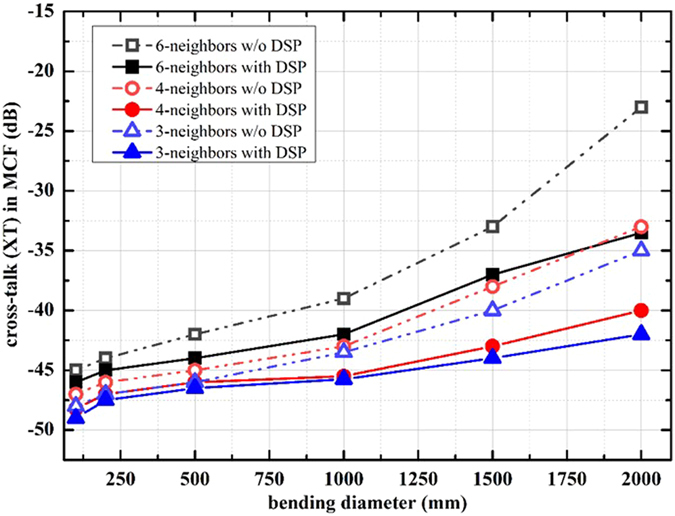
Performance of IXTC post-processing in the presence of bending in MCFs.

In addition to the factors discussed previously, another practical aspect for long haul communication is the bending effect on these kind of multiple core fibers. The analysis based on the coupled mode theory regarding bending and twisting is taken into consideration to quantify the bending diameter impact on XT. We have investigated identical model as previously to measure the impact of bending on our proposed receiver architecture on a single span of MCF and bending degradation effect is considered distributed on the whole length. The analytical results are reported in Fig. 7. We have considered cores with 6-, 4- and 3-neighbors and bending diameter ranging from 100 mm to 2000 mm. The results clearly depict efficient receiver performance in the presence of strong bending. To summarize, the above analytical results confirmed that XT can have a huge impact on the transmission performance/distance achievable with MCFs and should be addressed to optimize the performance of MCF based communication systems. DSP gives us substantial flexibility and performance to be incorporated with standard coherent SDM transmission to suppress inter-core XT.

## Conclusion

We have investigated the impact of intra- and inter-core XT on multi-carrier signals in MCFs by considering the SD- or HD-FEC levels. We propose a novel unified receiver architecture that can jointly compensates optical Kerr effects, intra- and inter-core XT, efficiently improving the system performance by 4.82 dB of Q-improvement. Furthermore, by compensating the inter-core XT, we analytically achieved a momentous gain in transmission reach; increasing the maximum transmission distance from 480 km to 1208 km. Due to the digital post-processing, the XT induced Q-penalty can be suppressed to be less than 1 dB till the inter-core XT of −11.56 dB for DP-16QAM signals as compared to the previously reported results of −23.5 dB by only opting all-optical design methods that can be interpreted as ≥4 *μ*m reduction in Λ. To summarize, we can adopt different core arrangements and Λ to fabricate high-count MCFs and the benefits from digital signal processing can be harvested efficiently to make high capacity SDM long-haul MCF transmission a reality.

## Methods

### Digital backward propagation (DBP)

At the receiver, DSP module of DBP is implemented by using the reverse split-step Fourier (SSFM) solution on Manakov equations. Because of the random residual birefringence in the fibers, this simple equation can be used instead of the vectorial non-linear Schroedinger equation. Mathematically, these equations can be expressed as in Eqs 1 and 2.


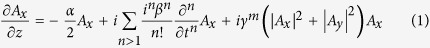



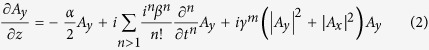


Whereas; *A*_*x*_ and *A*_*y*_ are the two polarization components of the total electric field, *α* is the attenuation constant of the fiber, *β* is the chromatic dispersion coefficient and *γ* is the non-linear parameter of the fiber^26^. The performance of DBP algorithm mainly depends on the estimation of propagating parameters of the above equations. The SSFM methods give us the flexibility to estimate these parameters with greater accuracy but at the expense of computational complexity. In this investigation we have used a A-SSFM algorithm with no split ratio of CD compensation^27^. Mathematically, the expression for A-SSFM can be given as in Eq. 3.


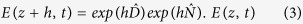


Whereas; 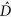 and 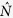 are linear and non-linear operators respectively. To further increase the performance efficiency of the algorithm, we have implemented a LPF with the non-linear step of DBP^59^. It is observed that during each DBP step intensity of the out-of-band distortion becomes higher. The distortion is produced by high-frequency intensity fluctuations modulating the outer sub-carriers in the non-linear sections of DBP. This limits the performance of DBP in the form of noise. To overcome this degradation factor, LPF is incorporated and the bandwidth of the LPF is optimized according to the number of DBP stages used to compensate the transmission impairments. The digital LPF not only limits the bandwidth of the compensating waveform so we can optimize the compensation for the low frequency intensity fluctuations without over-compensating for the high-frequency intensity fluctuations but also minimizes the required over sampling factor.

### Extended Kalman filter based adaptive MIMO equalization

After the linear and non-linear compensation of transmission impairments via DBP, Kalman filter based adaptive MIMO equalization is implemented. This DSP block compensates the XT in the neighboring sub-carriers and further enhances the system performance in terms of transmission reach, input power and non-linear tolerance^31^. This MIMO DSP block consists of signal rotator, finite impulse response (FIR) filter with tap co-efficients *h*_11_, *h*_12_, *h*_21_ and *h*_22_. In this paper, as in Fig. 2, we have implemented an extended Kalman fiter (EKF) based pre-rotation (PR) algorithm to estimate the state of polarization (SOP) for 2 × 2 MIMO processing per dual-carrier signal with greater accuracy and energy efficiency^63^. This facilitates reduced number of MIMO cross taps and avoids timing recovery failure due to differential group delay (DGD) at certain SOPs. While the FIR filters in MIMO DSP are responsible for the compensation of crosstalk in the sub-carriers, even a perfect estimate of inverse Jones matrix may not give the precise information about the sampling at the optimal points. Therefore, it is decisive to know the SOP of the signal in Stokes space (SS) ahead of MIMO filtering.

We implement the MIMO framework for *X* and *Y* polarizations of the incoming signal by taking into account the complex signals *X*_*in*_ and *Y*_*in*_ after CD compensation. We use real numbers *a*, *b*, *c* and *d* to represent the inverse Jones matrix (*J*_*PR*_). Mathematically, the updated output signals *X*_*out*_ and *Y*_*out*_ are given as in Eq. 4.


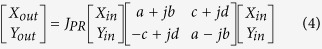


We initialize the EKF derivation by tracking the state vector *x*_*k*_ = [*abcd*]^*T*^. Whereas the update, *x*_*k*_, of the state variables can be given as in Eq. 5.


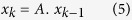


Where *A* is a 4 × 4 identity matrix. Two dimensional observation, *Z*_*k*_, is obtained having two functions, i.e. state variables (*x*_*k*_) and received signals (*X*_*out*_ and *Y*_*out*_). This non-linear measurement equation including the noise factor, *ξ*, can be given as in Eq. 6.


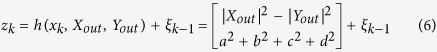


In summary, Eq. 6 is implemented to update the state variables for EKF based algorithm.

### Adaptive least mean square (LMS) equalizer

For the cancellation of inter-core XT, that is a linear and wavelength dependent phenomenon, we have implemented adaptive LMS equalizer as shown in Fig. 3. However, synchronization of the information across the cores is decisive for efficient performance. As this equalizer is the integrated part of the DSP module as enlisted in Fig. 2, so the optical/electrical path for each core after the fan-out (FO) needs to be the same length and digital sampling should be synchronized across all cores. After processing through the DSP blocks as depicted in Fig. 2, the adaptive LMS equalizer^60^ is applied across all the cores and *X*- and *Y* –polarizations. Whereas, the filter coefficients (*w*_11_, *w*_12_, *w*_13_ and so on) are jointly and adaptively updated based on LMS algorithm. Each filter coefficient *w*_*i*,*j*_ represents the weighted cross-talk from core 1 to 19. This coefficient is updated according to the following Eq. 7.


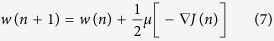


Where, 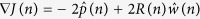, *R* and *p* are autocorrelation matrix and cross-correlation matrix, respectively.

### Wavelength dependent inter-core cross-talk (XT)

**Figure 8:**
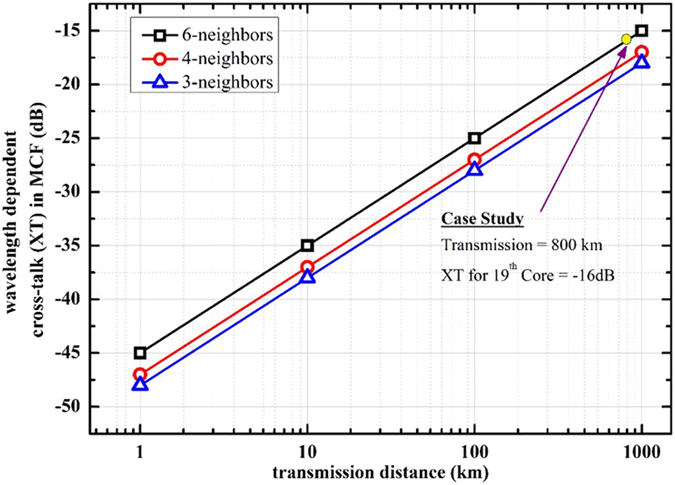
Analysis of inter-core XT as a function of transmission distance and number of neighboring cores.

The main issue with high capacity MCF transmission is how can we increase the number of cores (thus, total capacity) while keeping the inter-core XT low to allow error free and longer transmission distance since more dense cores (smaller core pitch ‘Λ’) normally increase the modes coupling among cores. As a first step, we compared the XT behavior of 19-core MCF in the wavelength region of 1550 nm. This is a standard telecommunication transmission window while it is also preferred for the downstream traffic signaling in the passive optical networks (PONs). The analytical expression for measuring the linear XT of the fiber is explained in^50^,^61^ and is wavelength dependent^64^. The refractive indices for the core, cladding and trench are *n*_1_, *n*_0_ and *n*_2_, respectively, where the refractive indices in the 1^*st*^ and 2^*nd*^ claddings in trench-assisted structure are considered same. The core radius, the distance from the outer edge of the 1^*st*^ cladding to the core center, the distance from the outer edge of trench to the core center and trench width are *a*_1_, *a*_2_, *a*_3_ and *w*_*tr*_, respectively. The relative refractive index differences between core and cladding, trench and cladding are Δ_1_ and Δ_2_, respectively. For formulation of XT in this paper, over a transmission length range of 1–1000 km, cladding diameter (CD) of 210 *μ*m and core-to-core distance of 35 *μ*m is used with the following values that are enlisted in Table 2. The results are plotted in Fig. 8.

## Additional Information

**How to cite this article**: Asif, R. Advanced and flexible multi-carrier receiver architecture for high-count multi-core fiber based space division multiplexed applications. *Sci. Rep.* **6**, 27465; doi: 10.1038/srep27465 (2016).

## Figures and Tables

**Table 1:** Parameters, number of cores and core geometries of different MCF types.

fiber Type	Structure	Core-to-Core Dist. (*μ*m)	Clad. Dia. (*μ*m)	Experiment/Theoretical*	Reference
**7-core**	single ring structure	48.5	230	51.1 Tbit/s QPSK, 2520 km	[44]
**12-core**	square lattice structure	43	230	20 WDM × 3 LP mode × 40 Gbit/s DP-QPSK, 527 km	[45]
**19-core**	double ring structure	62	318	2.05 Pbit/s, DP-64QAM, 9.8 km	[37]
**22-core**	double ring structure	41–48	260	2.15 Pbit/s, DP-64QAM, 31 km	[36]
**24-core**	square lattice structure	33.3	230	*32QAM, 100 km, XT measurements	[49]
**30-core**	ring/square lattice structure	30	230	*100 km, XT measurements	[51]
**36-core**	4-layer hexagonal lattice	34	306	403.7 Tbit/s, DP-QPSK, 5.5 km	[48]

**Table 2:** Physical parameters of MCF to evaluate XT at *λ* = 1550 nm.

Parameter	Value
n_*o*_	1.44
a_1_	4.0 *μ*m
Δ_1_	0.3%
a_2_	8.0 *μ*m
Δ_2_	−0.7%
w_*tr*_	4.0 *μ*m
Λ	35 *μ*m
CD	210 *μ*m
